# Adherence to Data-Driven Dietary Patterns and Lung Cancer Risk: A Systematic Review and Dose–Response Meta-Analysis

**DOI:** 10.3390/nu15204406

**Published:** 2023-10-17

**Authors:** Roberto Fabiani, Gianandrea La Porta, Laura Li Cavoli, Patrizia Rosignoli, Manuela Chiavarini

**Affiliations:** 1 Department of Chemistry, Biology and Biotechnology, University of Perugia, 06123 Perugia, Italy; roberto.fabiani@unipg.it (R.F.); gianandrea.laporta@unipg.it (G.L.P.);; 2 Department of Biomedical Sciences and Public Health, Section of Hygiene, Preventive Medicine and Public Health, Polytechnic University of the Marche Region, 60126 Ancona, Italy; m.chiavarini@staff.univpm.it

**Keywords:** lung cancer, dietary patterns, Western/meat, healthy/prudent, principle component analysis, dose–response meta-analysis

## Abstract

The effect of dietary patterns on lung cancer risk is currently debated. In this study, we evaluated the association between different “a posteriori” dietary patterns and lung cancer risk. The search was carried out (February 2023) through Scopus, Web of Science, and PubMed databases. Meta-analysis was performed by a random-effects model using risk values (RR and OR) extracted from the 12 selected studies. Two main dietary patterns were identified and named “Western/meat” and “Healthy/prudent”. The highest adherence to the “Western/meat” dietary pattern significantly increased the lung cancer risk (OR = 1.39; 95% CI: 1.17–1.65; *p* = 0.0002) while the highest adherence to the “Healthy/prudent” pattern reduced it (OR = 0.65; 95% CI: 0.51–0.83; *p* = 0.001). A linear trend between both dietary patterns and lung cancer risk was observed. However, a statistically significant inverse dose–response trend was found only for the “Healthy/prudent” dietary pattern (regression coefficient = −0.0031, *p* = 0.003). Subgroup analyses showed that the “Western/meat” pattern significantly increased the lung cancer risk in former (*n* = 4) (OR = 1.93, 95% CI: 1.11–3.36) and current smokers (*n* = 7) (OR = 1.35, 95% CI: 1.06–1.71). Similarly, the “Healthy/prudent” pattern exerts a protective effect on former (*n* = 4) (OR = 0.61, 95% CI: 0.44–0.85) and current smokers (*n* = 8) (OR = 0.64, 95% CI: 0.46–0.88). For both dietary patterns, no significant effect was observed on never-smokers.

## 1. Introduction

Despite the notable advances over the past decades in the prevention and treatment, lung cancer is still the most important cause of cancer death worldwide. In 2020, over 2.2 million new cases of lung cancer were diagnosed (11.7% of the total) with almost 1.8 million deaths (18% of the total) [1]. Moreover, because most new cases are generally discovered after metastasis has spread outside the lung, the five-year overall survival rate so far is quite low, at 19% [2]. Smoking remains the principal lung cancer risk factor. However, although the prevalence of smoking is decreasing, the incidence of lung cancer in non-smokers is increasing [2]. In particular, it has been estimated that many new cases of lung cancer are not related to smoking (15% in men and 53% in women) [2]. This evidence suggests the need to better understand the role played by other risk/preventive factors in the occurrence of this disease. Air pollution is certainly a factor influencing lung cancer, as demonstrated by numerous studies showing an increase in risk associated with exposure to airborne pollutants including particulate matter (PM_10_ and PM_2.5_) [3] and nitrogen dioxide [4]. In addition, both occupational exposures (asbestos, vinyl chloride) and residential radon exposure have been positively associated with lung cancer incidence and mortality [5,6].

Similarly to what was previously suggested for gastrointestinal cancers and cancer at other sites, dietary habits could deeply influence the occurrence and progression of lung cancer [7]. Indeed, several human studies have investigated the effect of specific dietary components on lung cancer risk [7]. Recent meta-analyses have shown significant preventive effects of the intake of fruits and vegetables [8], nuts [9], citrus fruits [10], fish, and polyunsaturated fatty acids [11], among others. On the other hand, a high intake of meat and processed meats raises the risk of lung cancer [12], while no significant effects have been observed for milk, dairy, and calcium intake [13]. However, rather than studying individual foods/nutrients, nutritional epidemiology in recent years has shifted to examining the effect of different dietary patterns on various chronic diseases, including cancer. This approach makes it possible to study the effect of diet as a whole under conditions closer to reality [14]. Two main methodological approaches are in use for the identification of dietary patterns defined as (1) “a priori” (hypothesis-driven), which is based on previously known health effects of dietary components, and (2) “a posteriori” (data-driven) which solely relies on dietary intake data of the studied population. Examples of a priori dietary patterns are the glycemic index, the dietary inflammatory index, and the Mediterranean diet score. The a posteriori approach uses statistical methods including PCA (principal component analysis) and CA (cluster analysis) to generate dietary patterns which are named in various ways (i.e., Healthy, Prudent, Western patterns) [14].

Several epidemiological studies have highlighted the relationship between different dietary patterns and the incidence/mortality of lung cancer. Regarding the a priori dietary patterns, a recent meta-analysis on the basis of nine studies reported a statistically significant 14% increment in lung cancer risk in association with a higher adherence to the glycemic index [15]. On the other hand, high adherence to the Mediterranean Diet was associated with a statistically significant 16% lower risk of lung cancer compared with low adherence [16]. Regarding the data-driven dietary patterns and lung cancer risk, in 2016 a meta-analysis conducted on eight studies was published showing that a “healthy dietary pattern” reduced lung cancer risk [17]. Since then, numerous studies have addressed this issue, also taking into consideration unhealthy diets, with mixed results.

The purpose of this systematic review and meta-analysis was to address the strength of evidence and provide a quantitative estimate of the association between different dietary patterns defined by “a posteriori” methods and lung cancer risk. We also analyzed the dose-dependent effect of the two identified diets, namely “Western/meat” and “Healthy/prudent” on lung cancer risk, and the differences between smokers, former smokers, and non-smokers.

## 2. Materials and Methods

The present investigation followed the standard procedure reported in the MOOSE guidelines (Meta-analysis Of Observational Studies in Epidemiology) [18]. The protocol of this study has been recorded in the International Prospective Register of Systematic Reviews (www.crd.york.ac.uk/PROSPERO/ (accessed on 28 February 2023), Registration No: CRD42023400492).

### 2.1. Systematic Search and Criteria for Selection

We conducted an extensive literature search, through February 2023, using the following databases: Scopus (https://www.scopus.com/ (accessed on 28 February 2023)), Web of Science (http://wokinfo.com/ (accessed on 28 February 2023)), and PubMed (http://www.ncbi.nlm.nih.gov/pubmed/ (accessed on 28 February 2023)). The PICO (Population, Intervention/exposure, Comparison, Outcome/event) framework was used to determine the eligibility of studies (Table S1, in the Supporting Information online). Relevant articles were searched using a combination of the following keywords: (“healthy diet” OR “Western diet” OR “dietary pattern” OR “dietary index” OR “diet index” OR “diet diversity” OR “dietary habit” OR “eating pattern” OR “diet quality” OR “nutrient pattern” OR “food pattern” OR “dietary score” OR “diet variety” OR “diet score” OR “Mediterranean diet” OR “eating index” OR “food score”) AND (cancer OR tumor OR adenoma OR “neoplastic disease” OR neoplasia OR neoplasm) AND (lung OR pulmonary OR respiratory). In addition, to identify additional relevant publications, we examined the reference lists of selected articles and recent reviewers (published in the last three years). In any case, reviews and pooled analyses, although important to obtain general information, were excluded from the selection. The following criteria were used to identify potential articles: (i) the study design should be case-control or prospective; (ii) the association between dietary patterns derived by “a posteriori” methods and lung cancer risk should be evaluated; (iii) odds ratio (OR), relative risk (RR), or hazard ratio (HR) estimates with 95% confidence intervals (Cis) should be provided. Intervention studies, pooled analysis, molecular studies, in vitro, and/or animal studies, reviews or meta-analyses, case studies, ecologic evaluations, and commentary, were excluded. If several publications from the same study were present, the ones with the largest number of subjects were selected. The selection evaluation and the data abstraction and quality assessment of each included article were independently carried out by two investigators. In the case of disagreements, discussion and consultation with a third author were adopted.

### 2.2. Data Extraction and Quality Assessment

The following information, from the selected studies, was extracted: last name of first author, study design and name, year and location of publication, characteristics of population (age, number of cases and controls, cohort size, and incident cases), and follow-up duration. In addition, we reported the methods used to identify dietary habits, pattern types, adherence scores (tertile, quartile, and quintile), OR/RR/HR (95% CI), *p*-value for the trend in dose–response analysis, and the matched or adjusted variables. In the case of multiple estimates, those that adjusted for the most confounding factors were selected.

The quality of studies was determined by the nine-star system on the basis of the Newcastle–Ottawa Scale method [19]. Higher quality studies received a score of nine, while a score ≥ 7 indicated the study with an acceptable quality. However, no study was excluded because of these quality criteria to avoid selection bias.

### 2.3. Statistical Analysis

We estimated the association between lung cancer risk and adherence to dietary patterns considering the highest versus the lowest level of scores. The meta-analysis was performed as if all types of ratio were Ors, and risk estimates (95% confidence intervals) were calculated using a random-effects model.

The analysis was restricted to the dietary patterns identified by “a posteriori” methods. For inclusion in the meta-analysis, only patterns sharing most foods with similar factor loading were considered. From twelve articles selected [20,21,22,23,24,25,26,27,28,29,30,31], we identified the two most common dietary patterns with a similar factor loading of principle components. One dietary pattern was named “Western/meat” and had a high loading of processed/red meat, sweets, eggs, and refined grains. The paper included labeled it as “Western” [20,21,27,28], “Westernized Traditional” [22], “Frugal pattern” [23], “American/Western” [24], “Animal product” [25], “High meat protein” [29], “Pork, processed meat, and potatoes” [30]. A second dietary pattern was named “Healthy/prudent” and was characterized by a high loading of vegetables, fruits, poultry, whole grains, and fish. These patterns were labeled as “Prudent” [20,21,22,27,28], “Fruits and vegetables” [23,24], “Vitamins and fiber” [25], “Healthy eating” [26], “Antioxidants” [29], “Salad vegetables” [30], and “Health high-fiber-low-fat” [31].

In addition, we carried out a two-stage meta-analysis to determine the dose–response trend across categories, assuming linear relationships. These estimations were performed with the “dosresmeta function” included in the dosresmeta package [32] available for the R statistical framework [33].

Heterogeneity between studies was determined by the chi-square-based Cochran’s Q statistic [34]. The I^2^ values were used to define the level of heterogeneity as follows: no heterogeneity (I^2^ = 0%–25%), moderate heterogeneity (I^2^ = 25%–50%), large heterogeneity (I^2^ = 50%–75%), and extreme heterogeneity (I^2^ = 75%–100%) [35]. Differences with *p* ≤ 0.05 (derived from two-sided statistical tests) were considered statistically significant.

The methods of Begg and Mazumdar and Egger et al. were used to investigate whether the meta-analysis was influenced by publication bias, as previously reported [36,37]. The funnel plot asymmetry was tested on the basis of the rank correlation between the effect estimates and their sampling variances, and it was considered asymmetric when the intercept of Egger’s regression line deviated from zero, with a *p*-value < 0.05. The analysis of sensitivity was used to reveal the robustness of combined effect estimates. One study in each turn was omitted to investigate the influence of a single study on the overall risk estimate. For the analysis, the statistical program ProMeta version 3.0 (IDoStatistics-Internovi, Cesena, Italy) was used.

## 3. Results

### 3.1. Study Selection, Characteristics, and Quality Assessment

**Figure 1:**
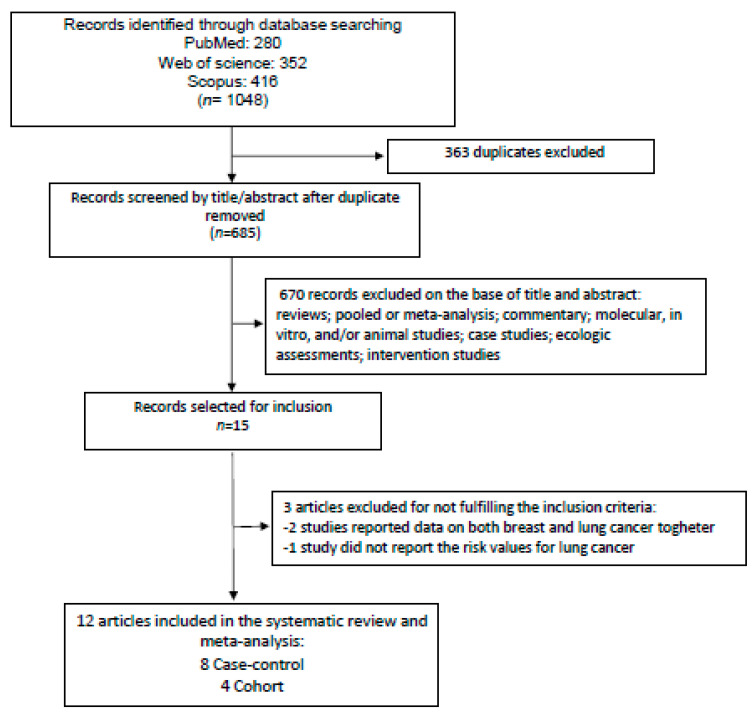
Diagram showing the search of the literature and selection of studies to be included in the meta-analysis.

A total of 1048 articles were identified from the initial search on three different databases (Scopus, Web of Science, PubMed). We removed 363 duplicates (Figure 1), leaving 685 articles for title and abstract analysis (Figure 1). In total, 670 papers were excluded from reading the title and abstract because they did not meet the PICO criteria for inclusion (Table S1, in the Supporting Information online). Fifteen articles remained for full-text analysis. Three items were excluded because they did not meet the criteria of inclusion. In particular, two studies reported the combined risk values for breast and lung cancer [38,39], and the other study did not show the lung cancer risk values [40]. Therefore, at the end, eight case-control studies [22,23,24,26,27,28,29,31] and four cohort studies [20,21,25,30] were selected to be included in the systematic review and meta-analysis after identification of the different dietary patterns (Figure 1).

Table 1 summarizes the properties including quality scores of the selected studies considering the lung cancer risk in association with adherence to “a posteriori” dietary patterns. Case-control studies (*n* = 8) were published between 2003 and 2020 and included a total of 6011 cases and 8263 controls. Only one study was population-based [23] while all others were hospital-based. The four cohort studies were published between 2005 and 2021, were carried out on a total of 505,665 subjects, and evidenced 3638 incident lung cancer cases. All investigations evaluated dietary habits by a food frequency questionnaire (FFQ) using from 20 to 201 different food items. In many cases, the FFQ was administered by the interviewer. Eleven studies used principle component analysis (PCA) to derive the “a posteriori” dietary patterns while one study used cluster analysis (CA) [31]. Two studies reported the lung cancer risk in association with two different dietary patterns [26,31], five studies considered three dietary patterns [20,21,22,24,29], four studies considered four dietary patterns [23,25,27,28], and one study considered five different dietary patterns [30].

Seven articles reported the lung cancer risk in women and men together [20,21,23,24,25,26,31], four studies were on men only [22,27,29,30] while only one study reported the risk separately for males and females [28]. Only two studies [23,24] evaluated the association between dietary patterns with different types of lung cancer (adenocarcinoma, squamous cell carcinoma, and others). Four studies were conducted in Europe [21,22,25,30], three in the USA [24,26,31] and Uruguay [27,28,29], and one each in Canada [20] and China [21].

All the selected papers reported dietary patterns suitable for inclusion in the “Healthy/prudent” pattern, while the “Western/meat” dietary pattern was not reported in one study [26]. In addition, although the study of Tsai et al. [31] included an unhealthy dietary pattern associated with the “Western/meat” pattern, it did not report the values of lung cancer risk. One study involved only never-smokers [26], while all others included smoking status among the adjustment variables. Some studies reported the risk on the basis of participants’ smoking status as follows: eight studies reported results on smokers [22,23,24,25,27,29,30,31], four on never-smokers [23,24,26,30], and four on former smokers [24,27,29,30].

In the last right column of Table 1 are shown the quality scores for each specific study based on the Newcastle–Ottawa Scale. In particular, the attribution of the scores is shown in supplementary Table S2 for case-control studies and Table S3 for cohort studies (available online). For case-control studies, the values of scores ranged from 6 to 9 (median: 8, mean ± SD: 7.8 ± 0.9) and seven studies reached high quality [22,24,26,27,28,29,31]. In the case of cohort design, three studies were of high quality [20,21,30].

### 3.2. Meta-Analysis

**Figure 2:**
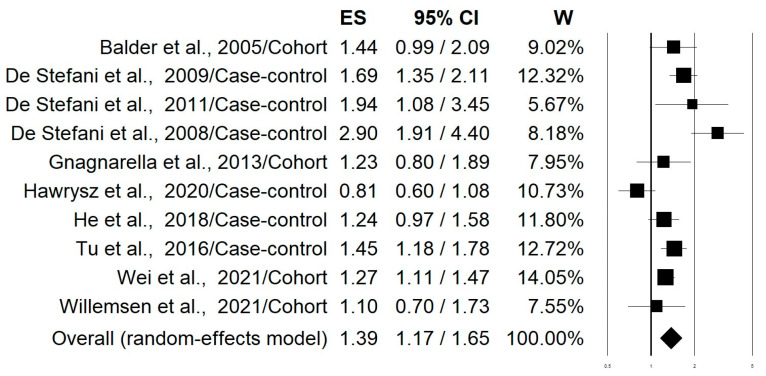
Forest plot of the studies that examined the associations of lung cancer risk with the highest versus lowest intake categories of the “Western/meat” dietary pattern [20,21,22,23,24,25,27,28,29,30].

**Figure 3:**
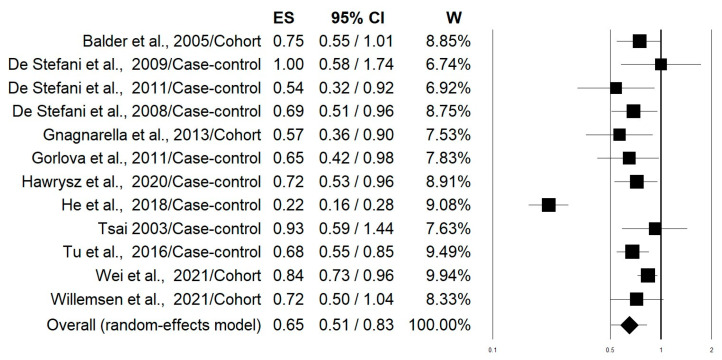
Forest plot reporting data on associations of lung cancer risk with the “Healthy/prudent” dietary pattern [20,21,22,23,24,25,26,27,28,29,30,31].

Figure 2 shows the forest plot of the 10 selected studies that examined the associations of lung cancer risk with the highest versus lowest intake categories of the “Western/meat” dietary pattern. The highest adherence to this dietary pattern significantly increased the lung cancer risk by 39% (OR = 1.39; 95% CI: 1.17–1.65; *p* = 0.0002). Stratifying the analysis on the basis of this study design slightly changed this effect with an increment in the risk of 50% and 27% for case-control and cohort studies, respectively (Table 2). The heterogeneity was rather high in both pooled data (I^2^: 72.45) and case-control studies (I^2^: 83.32), while it was not apparent in the cohort studies (I^2^: 00.00) (Table 2). Figure 3 shows the forest plot regarding the associations of lung cancer risk with the “Healthy/prudent” dietary pattern. Polling data from all 12 studies resulted in an evident and statistically significant 35% reduction in lung cancer risk associated with this dietary pattern (OR = 0.65; 95% CI: 0.51–0.83; *p* = 0.001). Analysis of the data separately for case-control (38% reduction) and cohort studies (21% reduction), produced essentially similar results (Table 2). In this case, the heterogeneity was also rather high in both pooled data (I^2^: 86.57) and in case-control studies (I^2^: 88.95), while there was no heterogeneity in the cohort studies (I^2^: 2.26) (Table 2). Regarding smoking status, subgroup analyses showed that the “Western/meat” pattern significantly increased the lung cancer risk in current smokers (*n* = 7) (OR = 1.35, 95% CI: 1.06–1.71) and former smokers (*n* = 4) (OR = 1.93, 95% CI: 1.11–3.36), while no statistically significant effect was observed on never-smokers (*n* = 4) (OR = 1.25, 95% CI: 0.80–1.93) (Table 2). Similarly, the “Healthy/prudent” pattern exerts a protective effect on current smokers (*n* = 8) (OR; 0.64, 95% CI: 0.46–0.88) and former smokers (*n* = 4) (OR; 0.61, 95% CI: 0.44–0.85), while no statistically significant effect was observed on never-smokers (*n* = 4) (OR; 0.60, 95% CI: 0.24–1.49) (Table 2). Because of the small amount of data, no further stratification according to gender, different types of lung cancer, and region was possible.

### 3.3. Dose–Response Analysis

**Figure 4:**
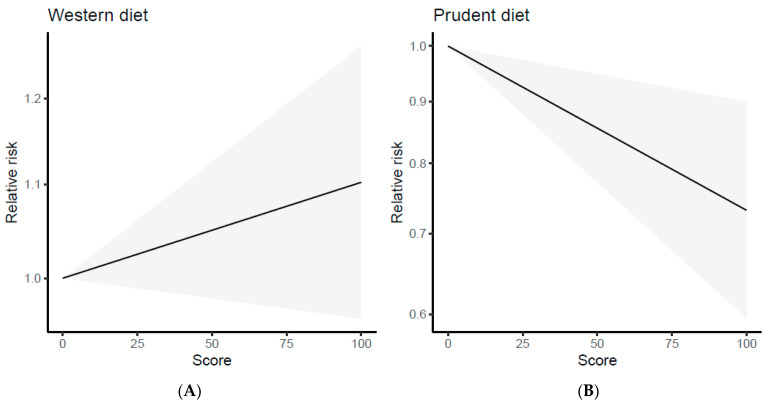
Dose–response plots of the linear relation between the intake of the “Western/meat” (**A**) and “Healthy/prudent” (**B**) dietary patterns and lung cancer risk.

The study of Tsai et al. [31] was excluded from this analysis because it did not report a dose-dependent effect. Therefore, the analysis was carried out on 11 papers, comprising 7 case-control studies [22,23,24,26,27,28,29] and 4 cohort studies [20,21,25,30]. Data regarding estimated trends in odds ratios (OR) based on dietary consumption are summarized in Supplementary Figure S1 (available online). The data on the “Western/meat” dietary pattern did not show a clear trend, as some studies indicated a higher risk of cancer with an increase in the percentile of dietary adherence, while others did not show any association. Hence, the linear dose–response curves suggested a slightly direct but not significant association between the “Western/meat” dietary pattern and cancer risk (regression coefficient = 0.0010, *p* = 0.169) (Figure 4A). The calculated risk is 1.001 (95% CI: 0.999–1.002), meaning that the risk of cancer would increase by 1.001 times when one unit dose of “Western/meat” dietary pattern is ingested. However, this was not statistically significant because the 95% CI includes 1.000. The results of the multivariate dose–response meta-analysis for the “Western/meat” dietary pattern showed no heterogeneity (Univariate Cochran Q-test: I^2^ = 0%, Q = 2.97, *p* = 0.89). The predicted lung cancer risk values for 20th, 50th, and 80th percentiles of adherence were 1.019 (95% CI: 0.992–1.048), 1.050 (95% CI: 0.980–1.125), and 1.081 (95% CI: 0.968–1.207), respectively. Instead, a statistically significant and inverse dose–response trend was found for the “Healthy/prudent” dietary pattern (regression coefficient = −0.0031, *p* = 0.003) as shown in Figure 4B. The risk of lung cancer varied by 0.997 (95% CI: 0.995–0.999; *p* < 0.01) for each percentile increment of this dietary pattern, with a statistically significant heterogeneity (Univariate Cochran Q-test I^2^ = 95.4%, Q = 151.55, *p* < 0.001). Furthermore, we did not observe significant differences in the sensitivity analysis when excluding one study at a time. The model predicted lung cancer risk values for the 20th, 50th, and 80th percentiles that were 0.940 (95% CI: 0.901–0.979), 0.855 (95% CI: 0.771–0.948) and 0.779 (95% CI: 0.660–0.918), respectively.

### 3.4. Publication Bias and Sensitivity Analysis

**Figure 5:**
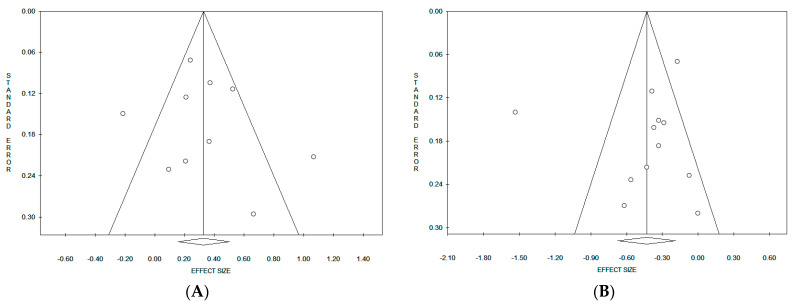
Funnel plots of the meta-analyses on the “Western/meat” (**A**) and “Healthy/prudent” (**B**) dietary patterns.

Considering the pooled data, on the basis of funnel plot symmetry for both “Western/meat” (Figure 5A) and “Healthy/prudent” (Figure 5B) dietary patterns, no evidence of publication bias was detected. Accordingly, the corresponding statistical evaluation by Egger’s and Begg’s tests resulted in the *p* value not being significant in both cases (Table 2). When results were stratified according to the study design, a significant publication bias was observed only in the cohort studies on “Healthy/prudent” dietary patterns by the Begg’s test (*p* = 0.042).

Removing each individual study for sensitivity analyses showed that the influence of a single study on lung cancer risk estimates was not substantially changed. Specifically, after eliminating the outlier study by He et al. [23] on the “Healthy/prudent” dietary pattern, a small change was found in the estimated risk (OR = 0.76; 95% CI: 0.69–0.82; *p* < 0.0001). It is noteworthy that the exclusion of this study from the analysis resulted in the disappearance of heterogeneity (I^2^ = 0.00, *p* = 0.531). Furthermore, lung cancer risk estimates calculated for the “Western/meat” dietary pattern varied from a value of 1.30 (95% CI: 1.13–1.51, *p* = 0.0004) when removing the study of De Stefani et al. 2008 [29] to 1.47 (95% CI: 1.26–1.72, *p* < 0.0001) when omitting the study of Hawrysz et al. [22].

## 4. Discussion

In this systematic review, all observational epidemiological studies showing the association between different “a posteriori” dietary patterns and lung cancer risk were identified. Twelve articles were selected and the two most common dietary patterns were evidenced: “Western/meat” and “Healthy/prudent”. Comparing the highest with the lowest intake categories, it was found that the “Western/carnivorous” dietary pattern was statistically significantly associated with a 39% increase in lung cancer risk, while maximum adherence to the “Healthy/prudent” dietary pattern resulted in a statistically significant 35% reduction in lung cancer risk. Furthermore, in the case of the “Healthy/prudent” dietary pattern, we found a statistically significant dose-dependent linear inverse correlation with lung cancer risk. Instead, for the “Western/meat” dietary pattern, we observed a linear dose-dependent positive correlation with lung cancer risk. However, this trend was not statistically significant.

Previous investigations support the hypothesis that different healthy and unhealthy dietary patterns can greatly influence chronic diseases, including cancer [41]. Indeed, in the last few years, several meta-analyses and systematic reviews have reported the relationship between data-driven dietary patterns and the risk of cancer in different organs including the bladder [42], colon and rectum [43], stomach [44], prostate [45], breast [46,47], and pancreas [48]. According to our results, all these studies showed a higher cancer risk associated with the Western dietary pattern.

Despite the different definitions used, the “Western/meat” dietary pattern is characterized by a high intake of red/processed meat, refined grains, and sugar-rich foods. All these dietary components may be reasonably involved in the carcinogenic properties of the Western diet. On the basis of the large amount of data on associations with colorectal cancer, red and processed meat have been classified by the IARC (International Agency for Research on Cancer) in 2015 as “probably carcinogenic” (Group 2A) and “carcinogenic” (Group 1) to humans, respectively [49]. Supporting this assumption, a recent meta-analysis showed a significant 24% (95% CI, 1.01–1.51) increment in lung cancer risk in non-smokers associated with high red meat consumption, although no effect was observed for processed meat [50]. The carcinogenic properties of meat could be mediated by suspected carcinogenic compounds, such as N-nitroso-compounds, heterocyclic aromatic amines, and polycyclic aromatic hydrocarbons. Most of these compounds are generated during meat processing or cooking at high temperatures [49]. Moreover, red and processed meats contain high amounts of saturated fat and heme iron, which can act as pro-oxidants and cause evident DNA damage [51]. The high amounts of refined grains in the “Western/meat” dietary pattern significantly reduce the intake of fiber, which may have a preventive effect on lung cancer. Indeed, a comprehensive prospective study investigating the role of the quality and quantity of carbohydrates on lung cancer appearance showed that higher intake of whole grains (RR:0.73; 95% CI 0.64–0.83) and dietary fiber (RR:0.62; 95% CI 0.54–0.72) reduced significantly the lung cancer risk [52]. In addition, it was also found that high intake of soft drinks (sugar-rich foods) increased the lung cancer risk by 23% (RR: 1.23; 95% CI 1.04–1.46) [52].

Regarding the lung cancer preventive ability of the “Healthy/prudent” dietary pattern, similar effects were evidenced also for cancers in other sites such as the colon [43], stomach [44], breast [47], and pancreas [48]. Instead, no significant association has been reported for both bladder [42] and prostate cancer [45]. The results of our study agree also with a previous meta-analysis, published in 2016 and conducted on eight studies, which showed that a healthy dietary pattern is associated with a reduced lung cancer risk (OR:0.81, 95%CI: 0.75–0.86) [17]. The main differences between the previous meta-analysis and our study are the following: (i) In the previous study, of the eight studies selected, two involved healthy dietary patterns identified by an “a priori” approach; (ii) our study included six new studies, of which five were published after 2016 [20,21,22,23,24] and one was excluded [27] from the previous meta-analysis; iii) in the previous study, a “Western/meat” dietary pattern was not identified and for the healthy diet, a dose-dependent effect was not reported [17].

The main characteristic of the “healthy/prudent” dietary pattern is related to the high intake of vegetables, fruits, and unrefined grains. These foods are rich in fiber, antioxidants, and anti-inflammatory compounds such as polyphenols, carotenoids, flavonoids, and vitamins, all of which can inhibit the process of carcinogenesis at different stages [53]. In particular, several experimental studies on animal models have investigated the chemopreventive effects of plant polyphenols for lung cancer [54]. Indeed, many isolated compounds, as well as complex extracts, have been demonstrated to be able to interfere with lung carcinogenetic processes including the xenobiotic metabolism, prevention of oxidative damage, and regulation of cell growth [54].

While writing this discussion, a meta-analysis was published on different dietary patterns and lung cancer risk [55]. Beyond the different pattern scores (Healthy Eating Index: HEI; Mediterranean diet index; Alternate HEI; Dietary Approaches to Stop Hypertension Index; Dietary Inflammatory Index), four main data-driven patterns were reported (Prudent pattern, Fruits/vegetables pattern, High meat/protein pattern, and Western pattern) and analyzed separately [55]. The data reported somewhat resemble our results, which were obtained by pooling both the “Prudent and Fruits/vegetables patterns” and the “High meat and Western patterns”. In the above study, neither stratification by smoking status nor dose–response effect was evaluated [55].

Subgroup analysis regarding the smoking status indicated that both dietary patterns identified in the present investigation were effective in modifying lung cancer risk in current and former smokers, while no significant association was found for never-smokers (Table 2). Although derived from a small number of studies, these results are particularly intriguing. They suggest that dietary habits may influence the lung cancer risk only in subjects that are, or have been, exposed to the carcinogens of the tobacco smoke. It is well known that smoking causes lung cancer in part through its pro-oxidant properties [56]. Therefore, it may be reasonable to assume that the “Healthy/prudent diet”, with its high amounts of antioxidants, is more effective in preventing lung cancer in smokers and less effective in non-smokers. Furthermore, it should be considered that there are many differences between lung cancer in smokers versus non-smokers. These multiple differences relate to histology, genetics, lifetime risk, and the role played by environmental risk factors [57]. It is possible that the lower lifetime risk of lung cancer, in addition to the greater role played by environmental factors in the etiology of lung cancer in never-smokers, may confound the results in this subgroup. Further studies are needed to clarify this aspect.

The data presented in our systematic review and meta-analysis have some strengths. We demonstrated a quantitative high and statistically significant association between data-driven dietary patterns and lung cancer risk. Sensitivity analysis suggests that the associations were robust because they remained significant even after individual studies were removed. In addition, a significant association was still evident after the stratification of data based on case-control and cohort study type. Most of the selected articles adjusted the risk estimate by considering several important factors that may have potential confounding capacity, including smoking, age, BMI, and physical activity. No evident publication bias was evidenced. Finally, we performed a dose–response analysis between adherence to the two different data-driven dietary patterns and lung cancer risk and investigated the shape of this association.

Nevertheless, due to various limitations, our data have to be interpreted with prudence. We noticed high heterogeneity, which could be related to the combination of surveys conducted with different methodological approaches and in different human populations. Due to the insufficient number of publications and the absence of necessary information in the original articles, it was not possible to make stratified analyses on the basis of important characteristics such as age, gender, ethnicity, etc. Overall, the meta-analysis used a low number of studies to calculate the risk and, although there was an evident consistency in the type of foods included under “Western/meat” and “Healthy/prudent” patterns, some variations in the categories of food consumption may still exist. In particular, regarding the “Western/meat” diet, insufficient information was produced in the articles on how and how much meat was processed. Furthermore, misclassification within the two dietary patterns identified could also be present because the principal component analysis is a subjective method, which may introduce variability along all different steps of dietary pattern identification [58]. Other constraints of this meta-analysis can be related to the fact that the included studies pooled data obtained directly from the population. In addition, each investigation presents its own weaknesses regarding the in-study design and classification of subjects.

## 5. Conclusions

Our findings suggest robust evidence that data-driven dietary patterns mainly “Western/meat” and “Healthy/prudent” patterns are able to deeply influence the lung cancer risk. This effect was particularly evident in smokers and former smokers. However, because of the small number of included studies, further prospective investigations of a larger number of subjects should be conducted to support this association in different subgroups regarding age, sex, different histological types of lung cancer, and ethnicity. In addition, further research should also consider possible interactions of dietary patterns with gut microbiota and genetic polymorphisms in relation to lung cancer risk.

## Figures and Tables

**Table 1:** Main characteristics of studies included in the systematic review and meta-analysis of dietary patterns (a posteriori) and lung cancer risk.

Author, Year Location	Study Design, Name, and Population Case/Control Follow-Up Incident Cases Age	Dietary Pattern Assessment and Identification Method	Dietary Pattern Type and Characteristics	Pattern Score	OR/RR (95% CI)	*p* for Trend	Matched or Adjusted Variables	NOS
Willemsen et al., 2021 [20] Canada	Cohort Alberta’s Tomorrow Project (ATP) 26,462 subjects Follow-up: 13.3 ± 3.3 years Incident cases: 252 Age: 35–69 years	124-item FFQ ^1^ 30 food groups PCA ^2^ Varimax rotation, EIG ^3^ > 0.35 Loading ≥ 0.35 3 factors, VE ^4^ 42.4% RRR ^5^ 4 factors, VE 88.3%	**PCA** **1. Western:** grain, non-whole grains, vegetables, white potatoes, cheese, lamb, pork, beef, luncheon meats (red and processed meats), discretionary fats, added sugar	Quartile 1 Quartile 2 Quartile 3 Quartile 4	1.00 (Ref.) 1.06 (0.74–1.52) 1.10 (0.76–1.59) 1.10 (0.70–1.73)	0.64	Age, sex, BMI ^6^, energy intake, smoking status, physical activity	9
			**2. Prudent:** vegetables, fruits, lean meat from fish and other sea food	Quartile 1 Quartile 2 Quartile 3 Quartile 4	1.00 (Ref.) 0.77 (0.55–1.07) 0.71 (0.50–1.01) 0.72 (0.50–1.04)	0.50		
			**3. Sugar, fruits, and dairy**: grain servings, especially whole grains, fruits, dairy, and teaspoons of added sugar	Quartile 1 Quartile 4	1.00 (Ref.) 0.67 (0.46–0.98)	0.007		
			**RRR** **1. Dietary fiber**: grain servings, vegetables, and fruits **2. vitamin D:** dairy, fish and other seafood **3. Fructose:** fruits and teaspoons of added sugar **4. Discretionary fat**: solid fats present within the “Milk” and “Meat and Beans	Quartile 1 Quartile 4 Quartile 1 Quartile 4 Quartile 1 Quartile 4 Quartile 1 Quartile 4	1.00 (Ref.) 0.66 (0.41–1.06) 1.00 (Ref.) 0.79 (0.55–1.13) 1.00 (Ref.) 1.54 (1.09–2.18) 1.00 (Ref.) 0.66 (0.44–0.98)	<0.0001 <0.0001 <0.0001 0.058		
Wei et al., 2021 [21] UK	Cohort UK Biobank 416,588 subjects Follow-up: 7.13 years Incident cases: 1782 Age: 40–69 years	FFQ/24 h dietary intake 16 food groups PCA Varimax rotation, EIG > 1 Loading ≥ 0.3 3 factors, VE 32%	**1. Western:** beef, lamb, mutton, pork and processed meat	Quartile 1 Quartile 2 Quartile 3 Quartile 4	1.00 (Ref.) 1.00 (0.87–1.16) 1.05 (0.91–1.21) 1.27 (1.11–1.46)		Age, sex, geographical region, smoking status, ethnicity	9
			**2. Prudent:** salad, raw vegetables, cooked vegetables, fresh fruit, dried fruit, oily fish, non-oily fish and water	Quartile 1 Quartile 2 Quartile 3 Quartile 4	1.00 (Ref.) 0.96 (0.84–1.09) 0.88 (0.77–1.00) 0.84 (0.73–0.96)			
			**3. Open sandwich:** processed meat, bread, tea and cheese	Quartile 1 Quartile 4	1.00 (Ref.) 1.08 (0.94–1.24)			
Hawrysz et al., 2020 [22] Poland	HB ^7^ case-control Cases: 187 Control: 252 Men Age: 45–80 years, mean 62.6 ± 7.2 years	62-item FFQ 23 food groups PCA Varimax rotation, EIG > 1.0 Loading > 0.3 3 factors, VE 31%	**1. Westernized Traditional:** red and processed meats, white meat, potatoes, other fats, vegetables, refined grain, sweetened beverages, energy drinks, sugar, honey, sweets	Tertile 1 Tertile 2 Tertile 3	1.0 (Ref.) 0.79 (0.45–1.37) 0.81 (0.60–1.08)		Age, BMI, smoking, socioeconomic status, physical, occurrence of lung cancer in relatives, occupational exposure in the workplace	8
			**2. Prudent:** whole grain, fruits, nuts, seeds, vegetables, fish, legumes, fruit, vegetable-fruit juices	Tertile 1 Tertile 2 Tertile 3	1.0 (Ref.) 0.63 (0.37–1.08) 0.72 (0.53–0.96)			
			**3. Sweet Dairy:** animal fats, milk, fermented and sweetened milk drinks and cheese, eggs, cheese, sugar, honey, sweets, breakfast cereals, refined grain products, vegetable oils, dried fruit and preserves	Tertile 1 Tertile 3	1.0 (Ref.) 0.99 (0.75–1.30)			
He et al., 2018 [23] Southeast China	PB ^8^ case-control Cases: 1166 Control: 1179 Age: mean 58.93 ± 15.44 years	20-item FFQ 11 food groups PCA Varimax rotation Loading > 0.4 4 factors, VE 49.53%	**1. Cereals/wheat and meat:** high quality protein, such as seafood, kelp and seaweed, egg and beans	Quartile 1 Quartile 4	1.0 (Ref.) 0.831 (0.645–1.070)	0.230	BMI, incomes, occupation, education, family history of lung cancer, history of lung diseases, environmental tobacco smoke, smoking status	6
			**2. Fruits and vegetables:** milk, fruits and vegetables	Quartile 1 Quartile 2 Quartile 3 Quartile 4	1.0 (Ref.) 0.447 (0.354–0.566) 0.285 (0.221–0.368) 0.216 (0.164–0.284)	<0.001		
			**3. Frugal pattern:** cereals/wheat and meat: pork, beef, lamb, poultry	Quartile 1 Quartile 2 Quartile 3 Quartile 4	1.0 (Ref.) 0.873 (0.675–1.129) 0.897 (0.695–1.159) 1.235 (0.966–1.581)	0.073		
			**4. High quality protein:** sweet potato and salty vegetables	Quartile 1 Quartile 4	1.0 (Ref.) 1.283 (0.999–1.643)	0.063		
Tu et al., 2016 [24] USA	HB case-control Cases: 2139 Age: mean 61.8 ± 10.4 years Control: 2163 Age: mean 61.9 ± 9.7 years	117-item FFQ 30 food groups PCA Varimax rotation, EIG > 1.0 Loading ≥ 0.38 3 factors, VE 26%	**1. American/Western**: hamburgers, cheeseburgers, French fries, fried potatoes, fried chicken, biscuits, rolls, chicken fried steak, gravies, pork chops, pork roasts, dinner, ham, bacons, sausage, chorizo, cheese dishes	Quintile 1 Quintile 2 Quintile 3 Quintile 4 Quintile 5	1.0 (Ref.) 1.02 (0.83–1.26) 1.10 (0.89–1.35) 1.33 (1.09–1.64) 1.45 (1.18–1.78)	<0.001	Age, sex, education, smoking status, pack-years, family history of lung cancer among 1° relatives, body mass index, physical activity, and total energy intake	9
			**2. Fruits and Vegetables**: deep yellow vegetables, cruciferous vegetables, dark leafy green vegetable, apples, pears, melons, tomatoes, grapes, strawberries, bananas, peaches	Quintile 1 Quintile 2 Quintile 3 Quintile 4 Quintile 5	1.0 (Ref.) 0.94 (0.77–1.14) 0.85 (0.69–1.03) 0.84 (0.68–1.03) 0.68 (0.55–0.85)	0.001		
			**3. Tex-Mex:** salsa, enchiladas, Spanish rice, refried beans, pinto beans, green chilis, jalapenos, serrano, peppers, avocado, guacamole, flour tortillas, soft tacos, flautas, crispy tacos, corn tortillas	Quintile 1 Quintile 5	1.0 (Ref.) 0.45 (0.37–0.56)	<0.001		
Gnagnarella et al., 2013 [25] Italy	Cohort COSMOS 4336 subjects Incident cases: 178 Follow-up: 5.7 years Heavy smokers	188-item FFQ 27 food groups PCA Varimax rotation, EIG > 1.0 Loading ≥ 0.63 4 factors, VE 81.38%	**1. Animal product:** animal protein, SFA, linoleic acid, Cholesterol, phosphorus, zinc, vitamin B2	Quartile 1 Quartile 2 Quartile 3 Quartile 4	1.00 (Ref.) 1.00 (0.64–1.56) 1.34 (0.88–2.04) 1.23 (0.80–1.89)	0.18	Baseline risk probability (age, sex, smoking history and exposure to asbestos) other nutrient patterns	6
			**2. Vitamins and fiber:** dietary fiber, potassium, vitamin C, total folate, b-Carotene, Vitamin E	Quartile 1 Quartile 2 Quartile 3 Quartile 4	1.00 (Ref.) 0.96 (0.66–1.41) 0.82 (0.55–1.22) 0.57 (0.36–0.90)	0.01		
			**3. Starch-rich:** vegetable protein, starch, sodium	Quartile 1 Quartile 4	1.00 (Ref.) 1.00 (0.66–1.51)	0.94		
			**4. Other PUFA**: other PUFA, vitamin D	Quartile 1 Quartile 4	1.00 (Ref.) 0.88 (0.58–1.34)	0.59		
Gorlova et al., 2011 [26] USA	HB case-control Cases: 299 Age: mean 61.52 ± 13.1 years Control: 317 Age: mean 61.53 ± 12.62 years Never smokers	201-item FFQ PCA Loading > 0.3 2 factors, VE 6.76%	**1. Mixed dishes:** onions raw/cooked, refried/pinto beans, spaghetti, lasagna, summer squash, cheese dishes without tomato souce, lettuce salad, green peas, avocado, guacamole, salsa, soft tacos, corn, including on the cob, Spanish rice, mayonnaise, grapes, dishes made with mole, raw tomatoes, boiled, baked, mashed potatoes, doughnuts, pastries, ketchup	Tertile 1 Tertile 3	1.00 (Ref.) 0.71 (0.41–1.19)		Age, gender, caloric intake, education Never Smokers	8
			**2. Healthy eating:** Low fat salad dressing, carrots, celery, broccoli, apples, applesauce, low fat yogurt, raw spinach, raw tomatoes, nonfat milk in cereal	Tertile 1 Tertile 2 Tertile 3	1.00 (Ref.) 0.95 (0.64–1.42) 0.65 (0.42–0.98)			
De Stefani et al., 2011 [27] Uruguay	HB case-control Cases: 200 Control: 800 Men Age: 30–79 years	64-item FFQ PCA Varimax rotation Loading > 0.39 4 factors, VE 37.4%	**1. Western:** red meat, processed meat, wine	Quartile 1 Quartile 2 Quartile 3 Quartile 4	1.0 (Ref.) 1.30 (0.73–1.32 1.73 (0.98–3.06 1.94 (1.08–3.45)	0.01	Age, residence, interviewer, hospital, education, family history of lung cancer, BMI, smoking, total energy intake	8
			**2. Prudent:** white meat, cheese, leafy vegetables, total fruits	Quartile 1 Quartile 2 Quartile 3 Quartile 4	1.0 (Ref.) 0.77 (0.49–1.22 0.65 (0.40–1.05 0.54 (0.32–0.92)	0.01		
			**3. Starchy vegetables:** vegetables potato, sweet potato, winter squash	Quartile 1 Quartile 4	1.0 (Ref.) 0.49 (0.28–0.86)	0.007		
			**4. Milk/coffee:** whole milk, coffee	Quartile 1 Quartile 4	1.0 (Ref.) 2.30 (1.35–3.90)	0.0002		
De Stefani et al., 2009 [28] Uruguay	HB case-control Cases: 920 Control: 2532 Age: mean 58/66 years	64-item FFQ PCA Varimax rotation Loading > 0.39 4 factors, VE 37.8%	**1. Western:** fried red meat, barbecue and eggs	Tertile 1 Tertile 2 Tertile 3	1.0 (Ref.) 1.23 (0.98–1.54) 1.69 (1.35–2.11)		Age, residence, urban/rural status, education, BMI, smoking, total energy intake, all the dietary patterns	8
			**2. Prudent:** poultry, fish, fresh vegetables, and total fruits.	Tertile 1 Tertile 2 Tertile 3	1.0 (Ref.) 1.00 (0.56–1.77) 1.00 (0.58–1.74)			
			**3. Traditional**: total grains, all tubers, desserts, and dairy foods	Tertile 1 Tertile 3	1.0 (Ref.) 1.08 (0.82–1.42)			
			**4. Drinker:** beer, wine and hard liquor	Tertile 1 Tertile 3	1.0 (Ref.) 1.28 (1.03–1.59)			
De Stefani et al., 2008 [29] Uruguay	HB case-control Cases: 846 Control: 846 Men Age: 30–89 years	64-item FFQ PCA Varimax rotation Loading > 0.49 3 factors, VE 0,93%	**1. High-meat protein:** saturated fat, monounsaturated fat, linoleic acid, linolenic acid, cholesterol	Tertile 1 Tertile 2 Tertile 3	1.0 (Ref.) 1.61 (1.16–2.35) 2.90 (1.91–4.40)	<0.0001	Age, residence, urban/rural status, education, family history of lung cancer BMI, smoking, alcohol, mate consumption, total energy intake	8
			**2. Antioxidants:** glucose, fructose, carotenoids, vitamin C, Vitamin E, folate	Tertile 1 Tertile 2 Tertile 3	1.0 (Ref.) 0.66 (0.50–0.89) 0.69 (0.51–0.96)	0.02		
			**3. Carbohydrates:** Starch, dietary fiber, thiamine, riboflavine, sodium, iron	Tertile 1 Tertile 3	1.0 (Ref.) 1.04 (0.72–1.52)	0.86		
Balder et al., 2005 [30] Netherlands	Cohort Netherlands Cohort Study 58,279 subjects Men Incident cases: 1426 Age: 62.6 years Follow-up: 9.3 years	150-item FFQ 51 food groups PCA Varimax rotation, EIG > 1.0 Loading > 0.35 5 factors, VE 23%	**1. Salad vegetables:** Leaf vegetables, allium vegetables, tomatoes, mushrooms, rice, pasta, oil, wine	Quintile 1 Quintile 2 Quintile 3 Quintile 4 Quintile 5	1.0 (Ref.) 1.07 (0.81–1.40) 1.02 (0.77–1.35) 0.75 (0.56–1.01) 0.75 (0.55–1.01)	0.008	Age, total energy intake, smoking, higher vocational or university education, family history of lung cancer, physical activity	7
			**2. Cooked vegetables:** Legumes, cabbages, leaf vegetables, cooked leaf vegetables	Quintile 1 Quintile 5	1.0 (Ref.) 0.86 (0.63–1.16)	0.18		
			**3. Pork, processed meat and potatoes:** Potatoes and potato products, bread, crackers, pork, processed meat, low-fat margarine, coffee	Quintile 1 Quintile 2 Quintile 3 Quintile 4 Quintile 5	1.0 (Ref.) 1.18 (0.87–1.61) 1.32 (0.96–1.80) 1.24 (0.90–1.71) 1.44 (0.99–2.09)	0.08		
			**4. Sweet foods:** Strawberries, savory snacks, cakes, sweet breads, cookies, and biscuits, added sugar	Quintile 1 Quintile 5	1.0 (Ref.) 0.62 (0.43–0.89)	0.002		
			**5. Brown/white bread substitution:** Apples, pears, bread, crackers, brown/whole meal types	Quintile 1 Quintile 5	1.0 (Ref.) 0.89 (0.65–1.20)	0.18		
Tsai et al., 2003 [31] USA	HB Case-control Cases: 254 Control: 184 Age: mean 63.13 ± 9.26 years	61-item FFQ Cluster analysis 2 factors	**1. Unhealthy hight-fat low-fiber**: alcohol, animal protein, saturated fat and cholesterol				Sex, age, smoking	7
			**2. Healthy high-fiber-low-fat:** carbohydrates, dietary fiber (folate, carotene, vitamin A, calcium, magnesium, potassium, copper)		1.0 (Ref.) 0.93 (0.59–1.44)			

^1^ Food Frequency Questionnaire; ^2^ Principal Component Analysis; ^3^ Eigenvalues; ^4^ Variance Explained; ^5^ Reduced Rank Regression; ^6^ Body Mass Index; ^7^ Hospital Based; ^8^ Population Based.

**Table 2:** Results of stratified analysis of the risk estimates for the highest compared with the lowest intake categories of different dietary patterns on the basis of study type and smoking status ^1,2^.

	Combined Risk Estimate		Test of Heterogeneity			Publication Bias	
	Value (95% CI)	*p*	Q	I^2^ %	*p*	*p* (Egger test)	*p* (Begg test)
	“Western/meat” dietary pattern						
Study type							
Case-control (*n* = 6) ^3^	1.50 (1.12–2.00)	0.006	29.98	83.32	<0.0001	0.583	0.851
Cohort (*n* = 4)	1.27 (1.13–1.43)	0.0001	0.84	0.00	0.839	0.869	0.174
Pooled ^4^ (*n* = 10)	1.39 (1.17–1.65)	0.0002	32.66	72.45	0.0001	0.580	0.655
Smoking status							
Current smokers (*n* = 7)	1.35 (1.06–1.71)	0.015	16.70	64.06	0.01	0.587	0.881
Former smokers (*n* = 4)	1.93 (1.11–3.36)	0.019	26.41	88.64	<0.0001	0.380	0.174
Never smokers (*n* = 3)	1.25 (0.80–1.93)	0.325	7.77	74.27	0.021	0.398	0.602
	“Healthy/Prudent” dietary pattern						
Study type							
Case-control (*n* = 8)	0.62 (0.43–0.89)	0.010	63.34	88.95	<0.0001	0.528	0.805
Cohort (*n* = 4)	0.79 (0.70–0.89)	0.0001	3.07	2.26	0.381	0.051	0.042
Pooled ^4^ (*n* = 12)	0.65 (0.51–0.83)	0.001	81.93	86.57	<0.0001	0.555	0.583
Smoking status							
Current smokers (*n* = 8)	0.64 (0.46–0.88)	0.007	43.33	83.85	<0.0001	0.156	0.805
Former smokers (*n* = 4)	0.61 (0.44–0.85)	0.003	8.39	64.25	0.039	0.241	0.497
Never smokers (*n* = 4)	0.60 (0.24–1.49)	0.266	71.17	95.78	<0.0001	0.464	0.999

^1^ The analysis was performed when a number of data ≥ 3 were available; ^2^ The risk estimates ware calculated using the random-effect model; ^3^ In brackets are indicated the number of articles included in the analysis; ^4^ Analysis was performed on case-control and cohort studies combined together.

## Data Availability

The data presented in this study are available on request from the corresponding author.

## Supplementary Materials

The following supporting information can be downloaded at: https://www.mdpi.com/article/10.3390/nu15204406/s1, Table S1: PICO criteria for inclusion of studies, Table S2: Methodological quality of case-control studies included in the meta-analysis, Table S3: Methodological quality of cohort studies included in the meta-analysis, Figure S1: Dose–response plots of the relation between the intake of the ”Western/meat” dietary pattern (left) and “Healthy/prudent” dietary pattern (right) and lung cancer risk in the different studies included in the meta-analysis.
