# Supplementary material for: Adherence to Data-Driven Dietary Patterns and Lung Cancer Risk: A Systematic Review and Dose–Response Meta-Analysis
Source: Nutrients. 2023 Oct 17;15(20):4406. doi: 10.3390/nu15204406 (PMC10610272; doi:10.3390/nu15204406)
Supplement: Supplementary file 1 [file nutrients-15-04406-s001.zip › nutrients-2636507-supplementary.pdf]

Supplementary Materials: Adherence to Data-Driven Dietary Patterns and Lung Cancer Risk: A Systematic Review and Dose–Response Meta-Analysis

Table S1. PICO criteria for inclusion of studies

Parameter	Criterion
Patients	Healthy adult participants or adult patients with lung cancer
Exposure	Dietary patterns identified by "a posteriori" methods
Comparison	Highest "a posteriori" dietary patterns adherence *vs* lowest "a posteriori" dietary patterns adherence
Outcome	"lung cancer" OR "respiratory cancer" OR "pulmonary cancer"

Table S2. Methodological quality of case-control studies included in the meta-analysis *.

First Author, Publication Year [Reference]	Adequate Definition of Cases	Representativeness of Cases	Selection of Control Subjects	Definition of Control Subjects	Control for Important Factor or Additional Factor †	Exposure Assessment	Same Method of Ascertainment for All Subjects	Non Response Rate ‡	Total Quality Scores
Hawrysz, 2020 [**22**]	✸	✸	✸	✸	✸	✸	✸	✸	8
He, 2018 [**23**]	---	✸	✸	---	✸	✸	✸	✸	6
Tu, 2016 [**24**]	✸	✸	✸	✸	✸✸	✸	✸	✸	9
Gorlova, 2011 [**26**]	✸	✸	✸	✸	✸	✸	✸	✸	8
De Stefani, 2011 [**27**]	✸	✸	✸	✸	✸	✸	✸	✸	8
De Stefani, 2009 [**28**]	✸	✸	✸	✸	✸✸	✸	✸	---	8
De Stefani, 2008 [**29**]	✸	✸	✸	✸	✸	✸	✸	✸	8
Tsai, 2003 [**31**]	✸	✸	✸	✸	✸	✸	✸	---	7

* A study could be awarded a maximum of one star for each item except for the item Control for important factor or additional factor. † A maximum of 2 stars could be awarded for this item. Studies that controlled for age received one star, whereas studies that controlled for other important confounders (smoking and total energy intake/BMI) received an additional star. ‡ One star was assigned if there was no significant difference in the response rate between control subjects and cases by using the chi-square test ($p < 0.05$).

Table S3. Methodological quality of cohort studies included in the meta-analysis *.

First Author, Publication Year [Reference]	Representativeness of the Exposed Cohort	Selection of the Unexposed Cohort	Ascertainment of Exposure	Outcome of Interest Not Present at Start of Study	Control for Important Factor or Additional Factor †	Assessment of Outcome	Follow-Up Long Enough for Outcomes to Occur ‡	Adequacy of Follow-Up of Cohorts §	Total Quality Scores
Willemsen, 2021 [**20**]	✸	✸	✸	✸	✸✸	✸	✸	✸	9
Wie, 2021 [**21**]	✸	✸	✸	✸	✸✸	✸	✸	✸	9
Gnagnarella, 2013 [**25**]	---	✸	✸	✸	✸	---	✸	✸	6
Balder, 2005 [**30**]	✸	✸	✸	---	✸	✸	✸	✸	7

* A study could be awarded a maximum of one star for each item except for the item Control for important factor or additional factor. † A maximum of 2 stars could be awarded for this item. Studies that controlled for age received one star, whereas studies that controlled for other important confounders (smoking and total energy intake/BMI) received an additional star. ‡ A cohort study with a follow-up time > 6 years was assigned one star. § A cohort study with a follow-up rate > 75% was assigned one star.

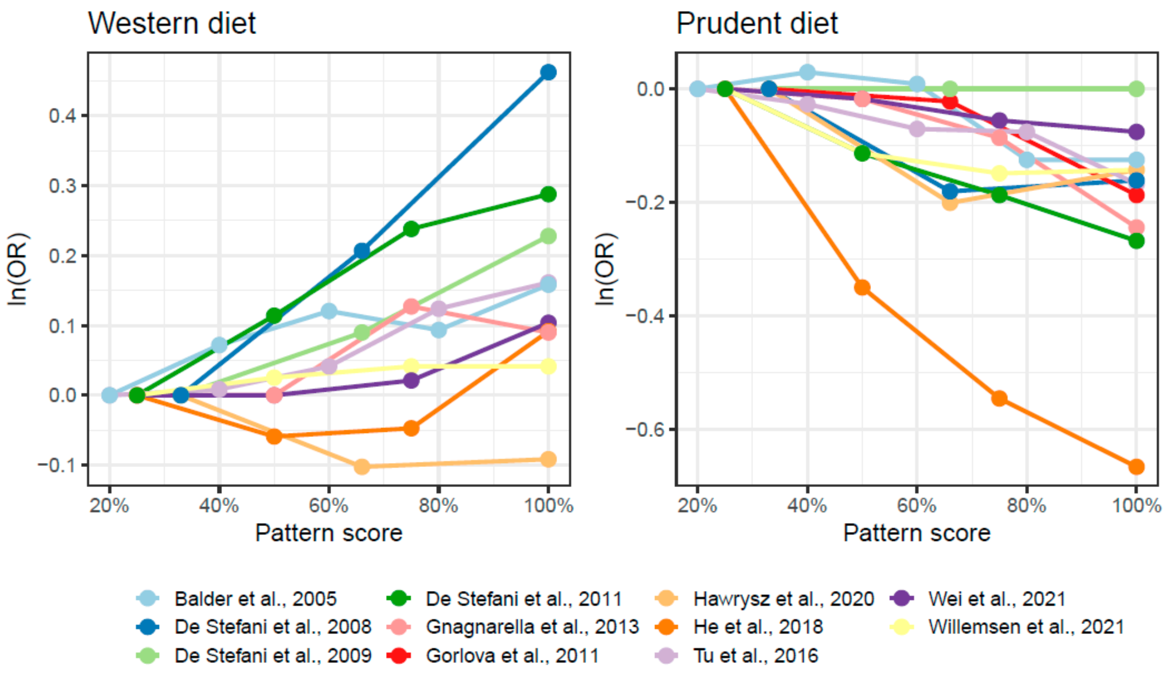

Figure S1. Dose-response plots of the relation between the intake of the ″Western/meat″ dietary pattern (left) and "Healthy/prudent" dietary pattern (right) and lung cancer risk in the different studies included in the meta-analysis [20-31].
